# Association of systemic immune-inflammation index with asthma and asthma-related events: a cross-sectional NHANES-based study

**DOI:** 10.3389/fmed.2024.1400484

**Published:** 2024-06-26

**Authors:** Tulei Tian, Meiling Xie, Gengyun Sun

**Affiliations:** ^1^Department of Respiratory and Critical Care Medicine, The First Affiliated Hospital of Anhui Medical University, Hefei, China; ^2^Department of Respiratory and Critical Care Medicine, The Affiliated Bozhou Hospital of Anhui Medical University, Bozhou, China; ^3^Bengbu Medical University Graduate School, Bengbu, China

**Keywords:** asthma, asthma-related events, systemic immune-inflammation index (SII), cross-sectional study, National Health and Nutrition Examination Survey (NHANES)

## Abstract

**Background:**

Asthma is associated with persistent airway inflammation, and numerous studies have investigated inflammatory markers causing asthma. However, the systemic immune-inflammation index (SII) is a novel inflammatory marker, with scarce research reporting on the correlation between SII and asthma and asthma-related events.

**Objective:**

The purpose of this study was to assess the relationship between SII and asthma and asthma-related events (including whether asthma is still present, asthma flare-ups in the past year, and asthma duration) using data from the National Health and Nutrition Examination Survey (NHANES).

**Methods:**

The study utilized data from NHANES 2009–2018 with asthma and asthma-related events as dependent variables and SII as an independent variable. Multifactor logistic regression was employed to assess the correlation between the independent and dependent variables. Smoothed curve-fitting and threshold effect analyses were also carried out to determine the presence of non-linear relationships. Subgroup analyses were then performed to identify sensitive populations.

**Results:**

In this study, we analyzed data from 40,664 participants to elucidate the association between SII and asthma and its related events. The study findings indicated a positive correlation between SII and asthma, with a relative risk increase of 0.03% for asthma incidence per one percentage point increase in SII (OR = 1.0003, 95% CI: 1.0002, 1.0004). For individuals still suffering from asthma, higher SII also indicated a positive correlation with ongoing asthma (OR = 1.0004, 95% CI: 1.0001, 1.0006). However, no statistically significant association was observed between SII and asthma exacerbations within the following year (OR = 1.0001, *p* > 0.05). When considering the duration of asthma, we observed a slight positive correlation with SII (*β* = 0.0017, 95% CI: 0.0005, 0.0029). Additionally, a significant non-linear relationship between SII and asthma duration emerged at the threshold of 504.3 (*β* = 0.0031, 95% CI: 0.0014–0.0048, *p* = 0.0003). Subgroup analysis revealed a stronger correlation between SII and asthma in male patients (OR = 1.0004, 95% CI: 1.0002–1.0006) and individuals aged 60 and above (OR = 1.0005, 95% CI: 1.0003–1.0007). No gender differences were observed for individuals still suffering from asthma. However, the positive correlation between SII and asthma was more pronounced in participants under 20 years old (OR = 1.0004 in Model 3, 95% CI: 1.0002–1.0006). Specific sensitive subgroups for asthma exacerbation recurrence within the past year were not identified. When considering asthma duration, we observed this association to be significant in male individuals (*β* = 0.0031 in Model 3, 95% CI: 0.0014–0.0049) as well as individuals aged 20 to 39 (*β* = 0.0023 in Model 3, 95% CI: 0.0005–0.0040).

**Conclusion:**

Our study concludes that SII is positively correlated with the persistence of asthma yet has limited predictive power for asthma recurrence. This highlights SII’s potential as a tool for assessing asthma risk and formulating targeted management strategies.

## Introduction

Asthma is a heterogeneous disease, usually characterized by chronic airway inflammation. It is defined by the history of respiratory symptoms, such as wheezing, shortness of breath, chest tightness, and cough, which vary over time and in intensity, together with variable expiratory airflow limitation (1). The disease substantially affects patients’ daily activities and long-term health, marked by frequent exacerbations, unstable treatment responses, and a variable disease trajectory. In 2019, asthma affected an estimated 262 million people and caused 455,000 deaths (2). As a result of its escalating global prevalence, asthma poses a daunting challenge to public health infrastructures (3, 4). Epidemiological evidence suggests that asthma is a primary culprit of mortality worldwide and a major economic encumbrance on healthcare systems (5). These factors highlight the critical need and immediacy for comprehensive research into asthma as well as the formulation of efficacious preventative and therapeutic approaches.

The systemic immune-inflammation index (SII) is recognized as a stable and accurate tool for reflecting both systemic and localized immune responses and the degree of inflammation. It mirrors the increase in absolute neutrophil and platelet counts, along with a decrease in lymphocyte count. Studies have uncovered a correlation between SII and the prognosis of various cancers (6–10). SII serves not only as a crucial predictor for adverse cardiovascular events in patients with ST-segment elevation myocardial infarction (STEMI) (11) but also as an essential biomarker indicative of heightened cardiovascular disease risk (12). Its potential value has been recently observed in research on chronic inflammatory diseases such as asthma (13–15). Asthma, characterized primarily by chronic airway inflammation and hyperresponsiveness, involves a close correlation between neutrophils and disease severity. Neutrophils exacerbate airway damage chiefly through the release of inflammatory mediators (16, 17). Concurrently, lymphocytes, particularly Th2 cells, play a pivotal role in modulating immune responses, influencing other immune cells through cytokine secretion to further boost chronic airway inflammation (18). Platelets also play a key role in asthma development, contributing to airway inflammation and hyperresponsiveness by participating in blood coagulation and inflammatory responses (19). Geng et al. (20) suggested that SII may surpass other inflammation indices, such as NLR and PLR, as it more comprehensively reflects the balance between inflammation and immune response. Therefore, in-depth investigations into the relationship between SII and asthma are of paramount importance. It can contribute to a better understanding of the pathogenesis of asthma, evaluating disease severity, and guiding treatment strategies.

However, there is currently a lack of large-scale population studies investigating the correlation of SII with asthma, and its related events such as asthma persistence, exacerbation frequency within the past year, and asthma duration. To address this gap in research, our study utilized NHANES data from 2009 to 2018 to explore the association between SII and asthma among adults in the United States. We aim to contribute to a deeper understanding of the pathogenesis of asthma, which could aid in evaluating asthma persistence, predicting exacerbation frequency, and formulating more effective treatment strategies.

## Materials and methods

### Data source

This study analyzed data from NHANES for the years 2009–2018. NHANES is administered by the National Center for Health Statistics (NCHS) and systematically assesses the health and nutritional status of a representative segment of the non-institutionalized civilian population in the United States. The data collection process employed a detailed, complex multi-stage probability group design, previously outlined in the literature (21). Following the STROBE guidelines and approved by the National Health Center’s Ethics Review Committee, all participants provided informed consent (22). The data used in this study are publicly available on the NHANES website.^1^

This study received approval from the Institutional Review Board (IRB) of the National Center for Health Statistics (NCHS), United States. All participants provided informed consent before undergoing the examination.

### Study design and population

**Figure 1:**
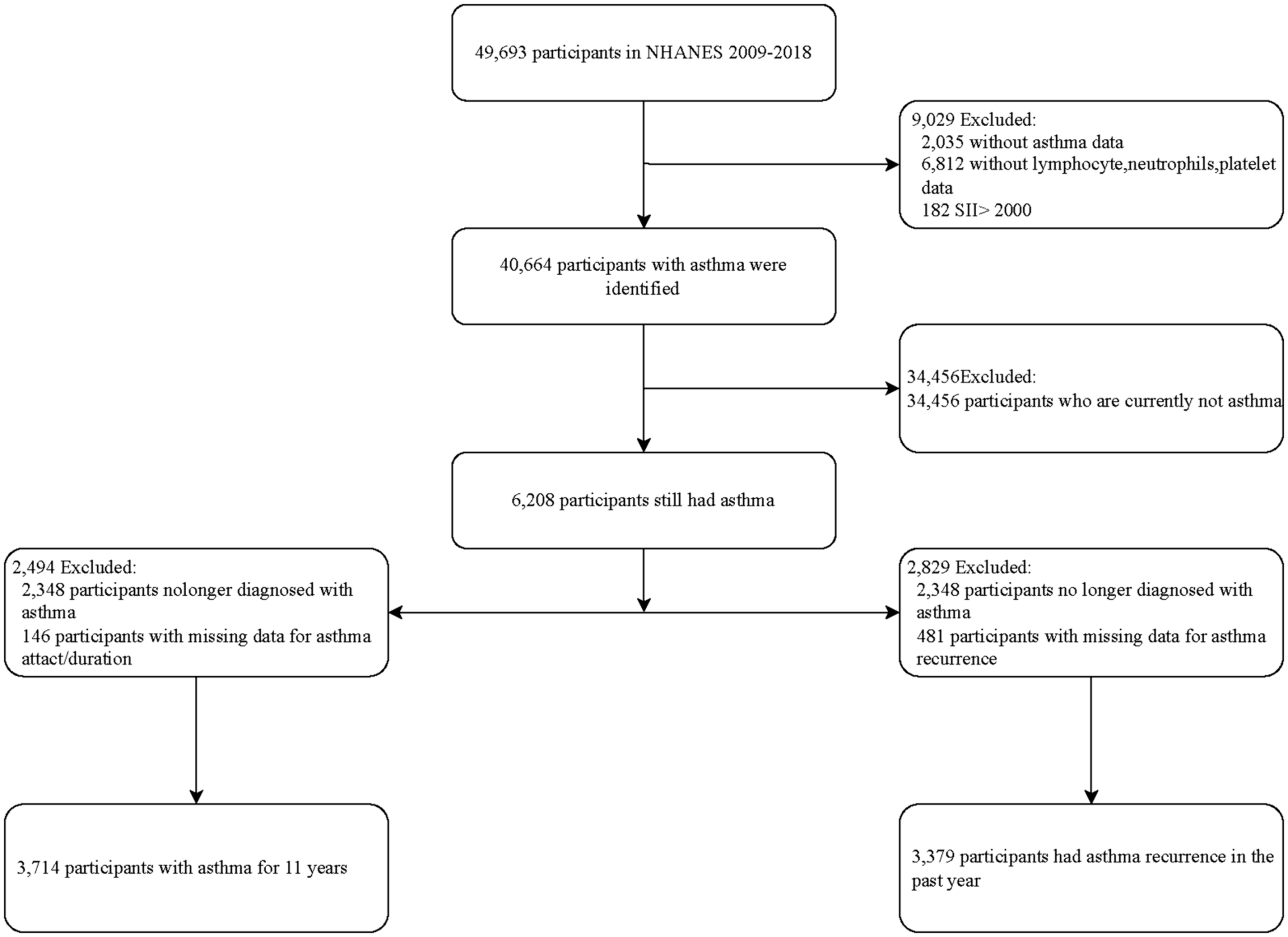
Flowchart of sample selection from the NHANES 2009–2018.

In this study, we analyzed the NHANES dataset from 2009 to 2018, which encompassed comprehensive asthma-related questionnaire data along with other necessary covariate information. The initial sample comprised 49,693 participants. Regarding inclusion criteria, we first excluded 2,035 participants lacking asthma information and 6,812 participants without lymphocyte (LY), neutrophil (NE), or platelet (PLT) data. Additionally, we analyzed the distribution of the SII and excluded 182 participants with abnormally high SII values (>2000, constituting less than 1% of the total sample) as detailed in Supplementary Table S1. Thus, the total number of participants included in the study examining the relationship between SII and asthma was 40,664. In further screening, we excluded 34,456 participants who were currently not diagnosed with asthma, ultimately including 6,208 participants for analysis of those still suffering from asthma. For subgroup analysis of asthma exacerbations within the past year, we excluded 2,348 participants no longer diagnosed with asthma and 481 participants lacking information on asthma exacerbations, resulting in 3,379 participants. Additionally, in the study of asthma duration, we excluded 2,348 participants no longer diagnosed with asthma and 146 participants lacking information on asthma onset/duration from the initial 6,208 participants, ultimately including 3,714 participants. The process for inclusion and exclusion is outlined in Figure 1.

### Definitions of SII

The SII, which includes platelet count, neutrophil count, and lymphocyte count, is calculated using the formula: SII = platelet count × neutrophil count/lymphocyte count. Blood samples were collected from fasting participants in the study, and complete blood cell count (CBC) was measured using an automated hematology analyzer (23).

### Definition of asthma and asthma-related events

Information regarding the prevalence of asthma and asthma-related events among the participants was sourced from a health status questionnaire. Participants were posed with the question, “Have you ever been told you have asthma.” Those who responded “yes” were classified as asthmatic. Regarding asthma-related events, first, respondents who answered “yes” to the self-administered question “Do you still have asthma?” were categorized as currently having asthma. Second, those who responded “yes” to either of the self-administered questions “Have you had an asthma attack in the past year?” or “Have you visited emergency care for asthma in the past year?” were defined as having experienced an asthma attack in the previous year. Finally, participants who could precisely respond to “At what age did you first have asthma?” were included in the study of asthma duration. These individuals with a history of asthma were listed as currently asthmatic. Overall, the duration of asthma was calculated as their current age minus the age at asthma onset.

### Covariates

Covariates included age, gender, race, educational level, and the Poverty Income Ratio (PIR), all derived from demographic variables. In analyzing survey questionnaire data, participants who affirmed having smoked at least 100 cigarettes in their lifetime were categorized as smokers. Their current smoking status was further classified into current, former, or never smokers, based on their response to the question “Do you currently smoke.” Those who answered affirmatively to “Have you had at least 12 alcoholic drinks in the past year?” were defined as alcohol consumers. The definitions of diabetes and hypertension were based on participants’ responses to “Have you been diagnosed with diabetes by a doctor?” and “Do you have hypertension?,” respectively. Due to limited information on participants’ use of corticosteroid medications, we adjusted our assessment of prescription medication usage, considering a positive response to “Have you used any prescription drugs in the past month?” as indicative of prescription drug use. Additionally, a positive response to “Does a close relative have asthma?” was taken as an indication of a family history of asthma. The study also collected information on participants’ activity levels, categorizing them as light, moderate, or vigorous based on the intensity of their work and recreational activities.

### Statistical analyses

In our study investigating the relationship between SII and asthma and its related events, all data processing and statistical analyses were conducted using R^2^ and EmpowerStats^3^. Normally distributed continuous variables were expressed as mean ± standard deviation, and comparisons between two groups were made using the U-test or *t*-test. Skewed continuous data were expressed as the median and interquartile range (IQR) and compared using the Kruskal–Wallis H-test. For missing data, the mean was used if less than 12% of the sample was missing; otherwise, continuous variables were categorized into “unclear groups” based on subgroups. Categorical or binary variables were expressed as counts (percentages) and compared using the chi-square test, with missing data defined as the “unclear group.”

To analyze the relationship between SII, asthma prevalence, and asthma-related events, multivariate logistic regression models were employed, considering covariates such as age, gender, race, education level, PIR, BMI, smoking status, alcohol consumption, activity intensity, prescription drug use, hypertension, diabetes, and family history of asthma. Three models were constructed to comprehensively evaluate the relationships between the independent and dependent variables: Model 1 was unadjusted; Model 2 adjusted for age, gender, and race; Model 3 adjusted for all covariates listed in Table 1. Notably, the age was not adjusted when asthma duration was the dependent variable.

**Table 1:** Characteristics of participants in asthma.

Characteristics	Non-asthma (*n* = 34,456)	Asthma (*n* = 6,208)	*p*-value
Age (years)	35.7881 ± 24.1826	33.6780 ± 22.8757	<0.001
Gender (%)			<0.001
Male	49.7533	47.3421	
Female	50.2467	52.6579	
Race (%)			<0.001
White	35.1405	36.5013	
Black	21.1052	27.5290	
Other race	43.7544	35.9697	
Education level (%)			<0.001
Less than high school	38.5071	42.0586	
High school	15.6896	15.2867	
More than high school	35.7674	36.3080	
Unclear	10.0360	6.3466	
PIR	2.3062 ± 1.5327	2.1343 ± 1.5302	<0.001
BMI (kg/m^2^)	26.1284 ± 7.5189	27.5943 ± 8.8091	<0.001
Alcohol (g), (%)			0.097
<0	64.1456	65.5284	
≥0	15.3268	14.9485	
Unclear	20.5276	19.5232	
Smoking (%)			<0.001
Now	12.5058	15.2706	
Ever	15.1759	15.5928	
Never	38.9947	33.3119	
Unclear	33.3237	35.8247	
Activity intensity (%)			<0.001
Vigorous	30.0064	32.3293	
Moderate	21.9178	21.7139	
Minor	24.1729	22.9543	
Unclear	23.9029	23.0026	
Prescribed medications (%)			<0.001
Yes	41.5196	60.4865	
No	58.4223	39.4491	
Unclear	0.0580	0.0644	
Hypertension, *n* (%)			<0.001
Yes	23.3109	26.5303	
No	48.1106	43.9916	
Unclear	28.5785	29.4781	
Diabetes (%)			<0.001
Yes	8.5587	10.4865	
No	89.6999	87.4034	
Borderline	1.6920	2.0457	
Unclear	0.0493	0.0644	
Close relative with asthma (%)			<0.001
Yes	17.0420	43.6372	
No	71.4012	47.0522	
Unclear	11.5568	9.3106	
WBC (1,000 cells/Ul)	7.2428 ± 3.1343	7.3977 ± 2.3735	<0.001
LY (1,000 cells/Ul)	2.4367 ± 2.3084	2.4009 ± 1.1782	0.232
NE (1,000 cells/Ul)	3.9834 ± 1.6841	4.1142 ± 1.7622	<0.001
PLT (1,000 cells/Ul)	253.3827 ± 67.9448	258.4328 ± 68.5331	<0.001
SII	462.2087 ± 262.6815	486.8242 ± 279.1511	<0.001

Mean ± SD for continuous variables: *p*-value was calculated by weighted linear regression model. % for categorical variables: *p*-value as calculated by weighted chi-square test.BMI, body mass index; PIR, poverty-income ratio; SII, systemic immune-inflammation index.

Participants were divided into four groups based on the quartile distribution of SII to assess whether the association between the independent variables and the incidence of asthma and related events demonstrated a trend with increasing SII (*p* for trend). Subgroup analyses were then conducted to identify sensitive populations in the relationship between SII and asthma prevalence, as well as related events. Finally, smooth curve-fitting and threshold effect analyses were performed. A *p*-value of <0.05 was considered statistically significant.

## Results

### Characteristics of participants

A total of 40,664 participants were enrolled in this study to investigate the association between SII and asthma onset. Table 1 lists the characteristics of the participants, of which 6,208 participants (15.3%) provided information related to asthma. Statistically significant differences were found between the asthmatic and non-asthmatic groups for all variables except drinking habits and lymphocyte counts (*p* < 0.001). Asthmatics were more likely to be female participants and have lower educational attainment, PIR, and higher body mass index (BMI) and SII values. Detailed characteristics of participants (Table 1) and characteristics of participants with asthma-related events are presented in the Supplementary Tables S2–S4.

### Association between SII and asthma and asthma-related events

**Figure 2:**
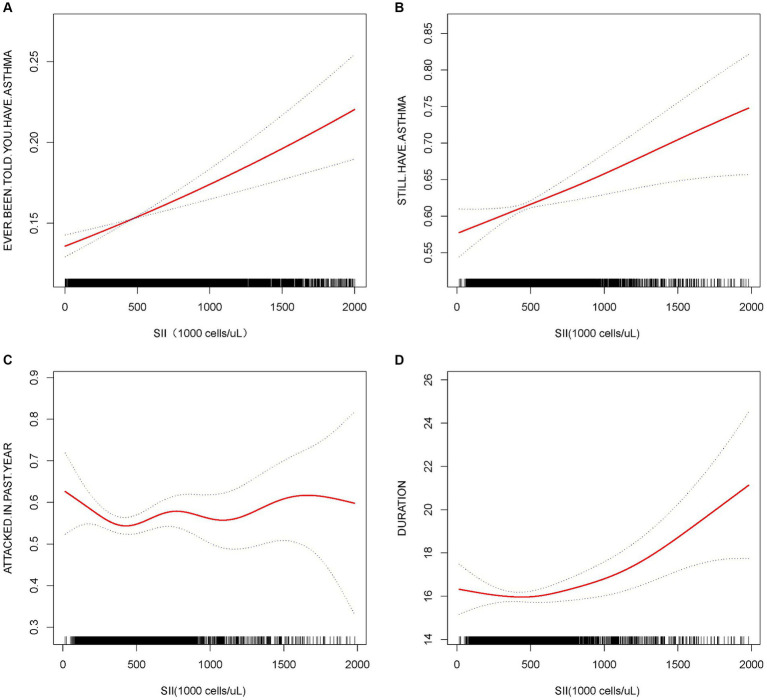
Association between SII and asthma and asthma-related. The solid red line represents the smooth curve fit between variables. The blue bands represent the 95% confidence interval from the fit. All the covariates were adjusted, but when considering the duration of asthma as the dependent variable, age was not adjusted.

Initially, we developed three models to examine the relationship between SII and asthma. In model 1 with SII as a continuous variable, after adjusting for the integrated model, we observed that the relative risk of asthma onset increased with each unit increase in SII (OR = 1.0003, 95% CI [1.0002, 1.0004]) and that the risk tended to elevate significantly as the level of SII increased (trend *p* < 0.05) (Table 2). Curve-fitting analysis showed that SII was positively associated with the development of asthma (Figure 2). Threshold effect analysis demonstrated a significant linear positive correlation between SII and asthma (*p* < 0.0001), and no significant non-linear threshold effect was detected (log-likelihood ratio *p* > 0.05) (Table 3).

**Table 2:** Multivariate regression of SII with asthma and related events separately.

Exposure	Model 1	Model 2	Model 3
	OR/*β* (95% CI)	OR/*β* (95% CI)	OR/*β* (95% CI)
ASTHMA			
SII (Continuous)	1.0003 (1.0002,1.0004)	1.0005 (1.0004,1.0006)	1.0003 (1.0002,1.0004)
Quartiles of SII			
Q1	Reference	Reference	Reference
Q2	1.1159 (1.0317,1.2070)	1.2159 (1.1227,1.3167)	1.1094 (1.0188,1.2080)
Q3	1.1489 (1.0625,1.2422)	1.2859 (1.1867,1.3935)	1.1232 (1.0290,1.2259)
Q4	1.2872 (1.1922,1.3899)	1.4633 (1.3506,1.5853)	1.2256 (1.1162,1.3457)
*p* for trend	<0.05	<0.05	<0.05
STILL.HAVE. ASTHMA	1.0003 (1.0001,1.0005)	1.0003 (1.0001,1.0005)	1.0004 (1.0001,1.0006)
SII (Continuous)			
Quartiles of SII			
Q1	Reference	Reference	Reference
Q2	0.9811 (0.8491,1.1338)	1.0110 (0.8724,1.1718)	1.0713 (0.9113,1.2593)
Q3	1.1324 (0.9788,1.3101)	1.1610 (0.9990,1.3493)	1.2260 (1.0369,1.4497)
Q4	1.1880 (1.0264,1.3751)	1.2048 (1.0334,1.4046)	1.2548 (1.0473,1.5035)
*p* for trend	<0.05	<0.05	<0.05
ATTACKED.IN.PAST. YEAR			
SII (Continuous)	0.9999 (0.9997,1.0001)	1.0000 (0.9997,1.0002)	1.0001 (0.9998,1.0003)
Quartiles of SII			
Q1	Reference	Reference	Reference
Q2	0.8181 (0.6744,0.9924)	0.8214 (0.6751,0.9994)	0.8978 (0.7291,1.1055)
Q3	0.7560 (0.6234,0.9168)	0.7546 (0.6189,0.9200)	0.8061 (0.6509,0.9983)
Q4	0.8982 (0.7401,1.0902)	0.9180 (0.7491,1.1251)	1.0167 (0.8059,1.2827)
*p* for trend	>0.05	>0.05	>0.05
DURATION			
SII (Continuous)	0.0066 (0.0049,0.0084)	0.0059 (0.0041,0.0077)	0.0017 (0.0005,0.0029)
Quartiles of SII			
Q1	Reference	Reference	Reference
Q2	3.1441 (1.7056,4.5825)	2.9634 (1.5206,4.4062)	0.0490 (−0.8596,0.9577)
Q3	4.5255 (3.0871,5.9640)	4.2495 (2.7920,5.7070)	−0.3404 (−1.2781, 0.597)
Q4	6.3671 (4.9290,7.8051)	5.7681 (4.2925,7.2438)	0.8900 (0.1235, 1.6563)
*p* for trend	<0.05	<0.05	<0.05

Model 1 = no covariates were adjusted.Model 2 = Model 1 + age, gender, and race were adjusted.Model 3 = All covariates were adjusted, but when considering the duration of asthma as the dependent variable, age was not adjusted.Use *β* value when analyzing the duration of asthma as the dependent variable.

**Table 3:** Threshold effect analysis of SII with asthma and STILL.HAVE. ASTHMA.

Outcome	ASTHMA	STILL.HAVE. ASTHMA
	OR (95% CI)	*p*-value OR (95% CI) *p*-value
Linear effect model	1.0003 (1.0002, 1.0004)	<0.0001 1.0004 (1.0001, 1.0006) 0.0022
Non-linear effect model		
Inflection point (*K*)	165.2258	909.0909
<*K*	0.9990 (0.9964, 1.0016)	0.4513 1.0002 (0.9999, 1.0005) 0.2405
≥*K*	1.0003 (1.0002, 1.0004)	<0.0001 1.0009 (1.0002, 1.0015) 0.0104
Log-likelihood ratio	0.3341	0.1063

Age, gender, race, education level, PIR, BMI, alcohol, smoking, activity intensity, prescription medications, hypertension, diabetes, and close relatives with asthma were adjusted in the models.

Subsequently, we examined the relationship between SII and STILL.HAVE. ASTHMA. In Model 3, each unit increase in SII was positively correlated with STILL.HAVE. ASTHMA (OR = 1.0004, 95% CI: 1.0001, 1.0006). Particularly when analyzing SII as a categorical variable by quartiles, individuals in the highest quartile demonstrated a 25.48% increased risk of STILL.HAVE. ASTHMA compared to those in the lowest quartile (Q4 vs. Q1 OR = 1.2548, 95% CI: 1.0473–1.5035), with risk increasing with higher levels of SII, as evidenced by a trend *p*-value less than 0.05 (Table 2). Curve fitting further demonstrated a positive correlation between SII and STILL.HAVE. ASTHMA, with an increased probability of STILL.HAVE. ASTHMA as SII levels rose (Figure 2). Threshold effect analysis revealed a dramatic elevation in STILL.HAVE. ASTHMA among individuals with higher SII levels (OR = 1.0009, *p* = 0.0104), particularly when SII exceeded the threshold of 909.09 (Table 3).

Moreover, regarding asthma recurrence within the past year, we observed no significant correlation between SII as a continuous variable and asthma recurrence in Model 3 (OR = 1.0001, *p* > 0.05), with quartile analysis and trend tests also exhibiting no significant association (*p* for trend >0.05) (Table 2). The curve-fitting outcomes indicated no clear linear relationship between increases in SII and the risk of asthma recurrence (Figure 2).

Finally, when considering the duration of asthma, we observed a modest positive correlation between SII and the duration of asthma after full model adjustment (*β* = 0.0017, 95% CI [0.0005, 0.0029]). Grouping SII by quartiles, the association with asthma duration strengthened with increasing SII levels, showing a statistically significant trend (*p* for trend <0.05) (Table 2). The curve fitting for the correlation between SII and asthma duration revealed a non-linear relationship (Figure 2). The subsequent threshold effect analysis indicated a significant non-linear relationship at the threshold point of 504.3 for SII. When the result was above this threshold, the association with asthma duration was significant (*β* = 0.0031, 95% CI: 0.0014–0.0048, *p* = 0.0003), while when the result was below this point, the association was not significant (*p* > 0.05) (Table 4).

**Table 4:** Threshold effect analysis of association between SII and duration.

Outcome	*β* (95% CI)	*p*-value
Non-linear effect model		
Inflection point	504.3	
<504.3	−0.0018 (−0.0049, 0.0013)	0.2530
≥504.3	0.0031 (0.0014, 0.0048)	0.0003
Log-likelihood ratio	0.0153	

Gender, race, education level, PIR, BMI, alcohol, smoking, activity intensity, prescription medications, hypertension, diabetes, and close relatives with asthma were adjusted in the models.

### Results of subgroup analysis

In subgroup analyses after stratification by gender and age, the association between SII and the onset of asthma was more significant in males (OR = 1.0004, 95% CI: 1.0002–1.0006 in Model 3) and in individuals aged 60 years and older (OR = 1.0005, 95% CI: 1.0003–1.0007 in Model 3). For the status of STILL.HAVE. ASTHMA, analyses did not reveal gender differences, but the positive correlation between SII and asthma status was more significant in younger participants under 20 years of age (OR = 1.0004, 95% CI: 1.0002–1.0006 in Model 3). Analysis of the recurrence of asthma attacks within the past year did not identify any specific sensitive subgroups. When considering the duration of asthma, male participants showed a significant positive association (*β* = 0.0031, 95% CI: 0.0014–0.0049 in model 3), and the association was also more pronounced in the 20- to 39 year-old age group (*β* = 0.0023 in model 3, 95% CI: 0.0005–0.0040) (Table 5).

**Table 5:** Subgroup analysis of SII and asthma and asthma-related events.

Characteristics	Model 1	Model 2	Model 3
	OR/*β* (95% CI)	OR/*β* (95% CI)	OR/*β* (95% CI)
ASTHMA			
Stratified by gender			
Male	1.0002 (1.0001, 1.0003)	1.0005 (1.0003, 1.0006)	1.0004 (1.0002, 1.0006)
Female	1.0005 (1.0003, 1.0006)	1.0005 (1.0004, 1.0006)	1.0002 (1.0001, 1.0004)
Stratified by age (years)			
<20	1.0004 (1.0002, 1.0006)	1.0006 (1.0004, 1.0008)	1.0003 (1.0001, 1.0005)
20–39	1.0003 (1.0001, 1.0005)	1.0003 (1.0001, 1.0005)	1.0002 (0.9999, 1.0005)
40–59	1.0005 (1.0003, 1.0007)	1.0004 (1.0002, 1.0006)	1.0002 (1.0000, 1.0005)
≥60	1.0004 (1.0002, 1.0006)	1.0005 (1.0003, 1.0007)	1.0004 (1.0002, 1.0006)
STILL.HAVE. ASTHMA			
Stratified by gender			
Male	1.0002 (1.0000, 1.0005)	1.0003 (1.0001, 1.0006)	1.0003 (1.0001, 1.0007)
Female	1.0003 (1.0000, 1.0005)	1.0004 (1.0001, 1.0006)	1.0003 (1.0001, 1.0007)
Stratified by age (years)			
<20	1.0005 (1.0003, 1.0007)	1.0004 (1.001, 1.0007)	1.0004 (1.0002, 1.0006)
20–39	1.0006 (1.0002, 1.0010)	1.0005 (1.0001, 1.0009)	1.0003 (0.9998, 1.0008)
40–59	1.0006 (1.0002, 1.0011)	1.0005 (1.0001, 1.0010)	1.0005 (0.9999, 1.0010)
≥60	1.0004 (1.0000, 1.0008)	1.0004 (1.0000, 1.0008)	1.0002 (0.9998, 1.0007)
ATTACKED.IN.PAST.YEAR			
Stratified by gender			
Male	0.9999 (0.9995, 1.0002)	1.0002 (0.9998, 1.0006)	1.0003 (0.9998, 1.0007)
Female	0.9999 (0.9996,1.0002)	0.9998 (0.9995,1.0001)	0.9999 (0.9995,1.0003)
Stratified by age (years)			
<20	0.9994 (0.9990, 0.9998)	0.9994 (0.9990, 0.9998)	0.9997 (0.9991, 1.0003)
20–39	1.0004 (0.9998, 1.0009)	1.0002 (0.9996, 1.0008)	1.0005 (0.9997, 1.0013)
40–59	0.9999 (0.9994, 1.0004)	0.9999 (0.9994, 1.0004)	0.9998 (0.9992, 1.0004)
≥60	1.0004 (0.9999, 1.0008)	1.0004 (0.9999, 1.0008)	1.0003 (0.9998, 1.0008)
DURATION			
Stratified by gender			
Male	0.0090 (0.0064, 0.0117)	0.0083 (0.0057, 0.0110)	0.0031 (0.0014, 0.0049)
Female	0.0042 (0.0019, 0.0066)	0.0040 (0.0016, 0.0064)	0.0003 (−0.0014, 0.0020)
Stratified by age (years)			
<20	0.0022 (0.0013, 0.0032)	0.0023 (0.0014, 0.0033)	0.0010 (0.0006, 0.0014)
20–39	0.0015 (−0.0039, 0.0069)	0.0005 (−0.0030, 0.0040)	0.0023 (0.0005, 0.0040)
40–59	0.0001 (−0.0039, 0.0041)	0.0007 (−0.0034, 0.0047)	0001 (−0.004, 0.0006)
≥60	0.0020 (−0.0066, 0.0106)	0.0019 (−0.0065, 0.0103)	00.0014 (0.0001, 0.0027)

Model 1 = no covariates were adjusted.Model 2 = Model 1 + age, gender, and race were adjusted.Model 3 = All covariates were adjusted, but when considering the duration of asthma as the dependent variable, age was not adjusted.The *β* value was utilized when analyzing the course of asthma as the dependent variable.

## Discussion

While prior research has dissected the association between SII and asthma (13, 24, 25), the intricacies of these relationships, especially the connection between SII and adverse events related to asthma, have yet to be fully elucidated. Our study sheds novel insights into the potential utility of SII in assessing asthma risk and guiding personalized management strategies. Our investigation further probes into the nexus between SII and both asthma and its associated events, substantiating that SII, as a marker of systemic inflammatory status, is intricately linked to an elevated risk of asthma incidence through a linear positive relationship with its abnormal elevation. Notably, our findings underscore that in individuals with elevated SII levels, there is a pronounced association with ongoing asthma conditions, highlighting the pivotal role of SII in the risk assessment of asthma. While the direct statistical significance between SII and the recurrence of asthma was not established, our analysis suggests that a positive correlation with the duration of asthma beyond a high SII threshold may indicate SII’s latent role in the pathophysiological advancement of asthma. Additionally, our demographic subgroup analysis accentuates the critical consideration of gender and age in the management of asthma, particularly highlighting the significance for male participants, older adults aged over 60, and youth under 20.

When juxtaposed with the extant body of literature, our study not only corroborates established findings but also elucidates novel insights. We reaffirmed the association of SII with asthma, aligning with prior investigations (13, 24, 25), and underscored the pivotal function of inflammatory markers within the asthma pathogenesis framework (26–28). Uniquely, our detailed analysis, segmented by gender and age, uncovers more granular distinctions in the relationship between SII and asthma, highlighting the previously underexplored impacts of gender and age specificity. This revelation advocates for the imperative of forthcoming research to intricately examine the moderating effects of gender and age on the dynamics between asthma and its inflammatory biomarkers. Furthermore, while existing studies (29, 30) postulate a potential linkage between inflammatory markers and asthma recurrence, our findings did not demonstrate a statistically significant linkage between SII and the recurrence of asthma. This observation indicates that the underpinnings of asthma recurrence are inherently multifaceted, being influenced by the confluence of diverse factors. It intimates the necessity for future inquiries to meticulously investigate how these elements synergistically influence the recurrence mechanism of asthma.

In this investigation, we meticulously analyzed SII’s correlation with asthma and its consequential events, underlining the pivotal roles of neutrophils, platelets, and lymphocytes in asthma’s pathogenesis. Our findings demonstrate that elevated SII levels accurately mirror the quantitative alterations in these inflammatory cells, thereby amplifying the risk of developing asthma. Notably, neutrophils intensify airway inflammation through the secretion of inflammatory mediators, such as IL-8 and TNF-α, with their effects markedly evident in cases of severe or refractory asthma (17, 31). Lymphocytes, particularly T cells, are instrumental in sustaining chronic inflammation in asthma by modulating eosinophil and other inflammatory cell activities via cytokines such as IL-4, IL-5, and IL-13 (32). Concurrently, allergens directly trigger platelet activation, leading to an upregulation of CD154 expression on platelets, a component involved in various immune responses, thereby facilitating the advancement of allergic asthma. This platelet activation is intricately linked to asthma exacerbation (33, 34). Moreover, our research indicates that an escalation in SII potentially enhances the vigor of inflammatory cells within the airways, precipitating airway hyperresponsiveness and structural alterations, such as smooth muscle proliferation and submucosal fibrosis, which aggravate asthma symptoms and elevate the risk of its persistence (35, 36). While a direct link between SII and recurrent asthma episodes was not definitively established, the observed trends suggest a possible association, underlining the necessity for larger cohort studies in future research to elaborate on these findings. Furthermore, the link between SII and the duration of asthma underscores the enduring effects of sustained inflammation on airway functionality and structure, accentuating the critical role of SII monitoring in asthma management, notably for evaluating inflammatory states, steering therapeutic strategies, and forecasting disease trajectory.

In this investigation, we meticulously examined the intricate association between SII and both asthma and its pertinent events (37), uncovering those particular demographics, notably male participants and individuals aged over 60, exhibit a pronounced positive correlation with SII levels and asthma onset. This underscores the utility of SII in gauging asthma risk within these subsets. Intriguingly, our analysis revealed an exceptional sensitivity to SII among adolescents under 20, potentially tied to the hormonal shift characteristic of puberty, with a particular emphasis on estrogen’s biological impacts. Estrogen’s capacity to simultaneously bolster immune responses and mitigate inflammatory reactions offers a plausible mechanism underlying observed disparities in asthma prevalence and SII variances across genders. This phenomenon intimates that estrogen might confer a protective effect against asthma in female participants by modulating immune and inflammatory dynamics, which may elucidate the observed lower prominence of SII in female asthma sufferers (38, 39). Additionally, age-related alterations in immune function may contribute to the observed associations between SII and asthma in different age groups. While the present study did not establish a direct link between SII and recurrent asthma episodes, it accentuates the imperative to incorporate individual traits, including gender, age, and hormonal levels, for a refined asthma risk assessment. Specifically, the role of estrogen in the pathogenesis of asthma merits additional exploration to elucidate the regulatory influence of sex hormones on asthma’s evolution, thereby informing the formulation of more efficacious therapeutic approaches.

Although this study has deepened our understanding of the relationship between SII and asthma, it is still subject to some limitations. First, the diagnosis of asthma in the NHANES database relies primarily on self-reported questionnaire surveys, which may not be as precise as medical diagnoses, potentially introducing recall bias that could affect the current findings. Second, due to the adoption of a cross-sectional study design, we cannot establish a causal relationship between SII and asthma. Causal inference is crucial for guiding clinical intervention measures. Third, although SII serves as a comprehensive indicator of inflammation and is easily measurable in clinical settings, incomplete data collection of neutrophil, lymphocyte, and platelet counts increases the risk of selection bias in the study results. Fourth, this study is limited to participants from the United States, and the results may not be generalizable to other populations with different risk factors and health behaviors. Furthermore, changes in asthma treatment strategies and the lack of detailed information on asthma medication usage and adherence during the study period may have influenced the observed associations between SII and asthma outcomes. Moreover, the potential confounding effects of unrecorded comorbidities on SII levels warrant further investigation in future studies. In light of these limitations, future research should consider adopting a longitudinal design. This would not only help ascertain the causal link between SII and asthma but also proffer more in-depth insights into the development and management of asthma, based on controlling for more potential confounding factors.

## Conclusion

Our study yielded a positive correlation between SII and the persistence of asthma, albeit with limited predictive power for recurrence. These findings underscore the potential of SII as a valuable tool in the identification of asthma risk and the development of targeted management strategies for individuals at heightened risk.

## Data availability statement

Publicly available datasets were analyzed in this study. This data can be found here: https://www.cdc.gov/nchs/nhanes/index.htm.

## Ethics statement

The studies involving humans were approved by Ethics Committee/Institutional Review Board: this study analyzed publicly available, anonymized data from the National Health and Nutrition Examination Survey (NHANES), which is conducted by the National Center for Health Statistics (NCHS), part of the Centers for Disease Control and Prevention (CDC). The NCHS Ethics Review Board has reviewed and approved the NHANES data collection protocol. The studies were conducted in accordance with the local legislation and institutional requirements. The participants provided their written informed consent to participate in this study. Written informed consent was obtained from the individual(s) for the publication of any potentially identifiable images or data included in this article.

## Author contributions

TT: Formal analysis, Funding acquisition, Software, Writing – original draft, Writing – review & editing. MX: Data curation, Validation, Visualization, Writing – review & editing. GS: Funding acquisition, Project administration, Resources, Supervision, Validation, Writing – review & editing.

## Footnotes

^1^http://www.cdc.gov/nchs/nhanes.htm

^2^http://www.R-project.org

^3^http://www.empowerstats.com
