# Supplementary material for: Association of systemic immune-inflammation index with asthma and asthma-related events: a cross-sectional NHANES-based study
Source: Front Med (Lausanne). 2024 Jun 26;11:1400484. doi: 10.3389/fmed.2024.1400484 (PMC11233796; doi:10.3389/fmed.2024.1400484)
Supplement: Supplementary file 1 [file Table_1.DOCX]

Supplementary Material

Supplement **Table 1** SII Outlier Removal

| Lower limit of range | Upper limit of range | Median of range | Frequency | Percentage (%) |
| --- | --- | --- | --- | --- |
| 0 | 2000 | 1000 | 40664 | 99.5544 |
| 2000 | 4000 | 3000 | 156 | 0.3819 |
| 4000 | 6000 | 5000 | 21 | 0.0514 |
| 6000 | 8000 | 7000 | 2 | 0.0049 |
| 8000 | 10000 | 9000 | 2 | 0.0049 |
| 10000 | 12000 | 11000 | 0 | 0.0000 |
| 12000 | 14000 | 13000 | 0 | 0.0000 |
| 14000 | 16000 | 15000 | 0 | 0.0000 |
| 16000 | 18000 | 17000 | 0 | 0.0000 |
| 18000 | 20000 | 19000 | 0 | 0.0000 |
| 20000 | 22000 | 21000 | 0 | 0.0000 |
| 22000 | 24000 | 23000 | 0 | 0.0000 |
| 24000 | 26000 | 25000 | 0 | 0.0000 |
| 26000 | 28000 | 27000 | 0 | 0.0000 |
| 28000 | 30000 | 29000 | 1 | 0.0024 |

Supplement **Table 2** Characteristics of participants in **STILL.HAVE. ASTHMA**

| Characteristics | No more asthma(n=2348) | Still have asthma(n=3764) | *P-value* |
| --- | --- | --- | --- |
| Age (years) | 33.2402 ± 21.2739 | 33.8167 ± 23.8184 | 0.2582 |
| Gender (%) |  |  | <0.001 |
| Male | 53.5775 | 43.3316 |  |
| Female | 46.4225 | 56.6684 |  |
| Race (%) |  |  | <0.001 |
| White | 37.2658 | 35.9989 |  |
| Black | 23.6371 | 29.9150 |  |
| Other race | 39.0971 | 34.0861 |  |
| Education level (%) |  |  | <0.001 |
| Less than high school | 39.4378 | 43.8629 |  |
| High school | 15.1193 | 15.1966 |  |
| More than high school | 41.7376 | 32.9171 |  |
| Unclear | 3.7053 | 8.0234 |  |
| PIR | 2.3532 ± 1.5886 | 1.9988 ± 1.4777 | <0.001 |
| BMI (kg/m2) | 27.0522 ± 7.9159 | 27.9328 ± 9.3357 | 0.012 |
| Alcohol (g), (%) |  |  | 0.004 |
| < 0 | 64.0545 | 66.5781 |  |
| ≥ 0 | 16.8654 | 13.7620 |  |
| Unclear | 19.0801 | 19.6599 |  |
| Smoking (%) |  |  | <0.001 |
| Now | 14.6082 | 15.4091 |  |
| Ever | 16.2266 | 15.0903 |  |
| Never | 37.0102 | 31.0574 |  |
| Unclear | 32.1550 | 38.4431 |  |
| Activity intensity (%) |  |  | <0.001 |
| Vigorous | 38.3305 | 28.6929 |  |
| Moderate | 22.4872 | 20.9617 |  |
| Minor | 20.6559 | 24.3624 |  |
| Unclear | 18.5264 | 25.9830 |  |
| Prescribed medications (%) |  |  | <0.001 |
| Yes | 44.6337 | 70.4038 |  |
| No | 55.3237 | 29.5165 |  |
| Unclear | 0.0426 | 0.0797 |  |
| Hypertension, n (%) |  |  | <0.001 |
| Yes | 23.3390 | 28.2678 |  |
| No | 52.1295 | 38.8417 |  |
| Unclear | 24.5315 | 32.8905 |  |
| Diabetes (%) |  |  | <0.001 |
| Yes | 7.5383 | 12.3273 |  |
| No | 91.0136 | 85.1488 |  |
| Borderline | 1.4480 | 2.4176 |  |
| Unclear | 0.0000 | 0.1063 |  |
| Close relative with asthma (%) |  |  | <0.001 |
| Yes | 36.9676 | 48.2200 |  |
| No | 56.5162 | 40.8342 |  |
| Unclear | 6.5162 | 10.9458 |  |
| WBC (1000 cells/Ul) | 7.1699 ± 2.2148 | 7.5490 ± 2.4608 | <0.001 |
| LY (1000 cells/Ul) | 2.3174 ± 1.0074 | 2.4562 ± 1.2759 | <0.001 |
| NE (1000 cells/Ul) | 4.0156 ± 1.6834 | 4.1788 ± 1.8094 | <0.001 |
| PLT (1000 cells/Ul) | 251.1955 ± 63.1633 | 263.2736 ± 71.3132 | <0.001 |
| SII | 472.2287 ± 259.3244 | 496.3067 ± 291.3586 | 0.015 |

Mean± SD for continuous variables: P value was calculated by weighted linear regression model. % For Categorical variables: P value as calculated by weighted chi-square test.

Supplement **Table 3** Characteristics of participants in **ATTACKED.IN.PAST. YEAR**

| Characteristics | Unattacked(n=1447) | Attacked(n=1902) | *P-value* |
| --- | --- | --- | --- |
| Age (years) | 35.6432 ± 23.6522 | 32.2482 ± 23.6589 | <0.001 |
| Gender (%) |  |  | 0.006 |
| Male | 45.8362 | 41.1146 |  |
| Female | 54.1638 | 58.8854 |  |
| Race (%) |  |  | 0.562 |
| White | 35.1388 | 34.8580 |  |
| Black | 31.2119 | 29.8633 |  |
| Other race | 33.6493 | 35.2787 |  |
| Education level (%) |  |  | <0.001 |
| Less than high school | 44.2112 | 42.5342 |  |
| High school | 17.8064 | 13.2492 |  |
| More than high school | 33.7170 | 33.2282 |  |
| Unclear | 4.2654 | 10.9884 |  |
| PIR | 2.0690 ± 1.4960 | 1.9480 ± 1.4593 | 0.011 |
| BMI (kg/m^2^) | 28.0876 ± 8.5476 | 27.8538 ± 9.9291 | 0.034 |
| Alcohol (g), (%) |  |  | 0.017 |
| < 0 | 64.7258 | 68.5068 |  |
| ≥ 0 | 15.0305 | 11.8822 |  |
| Unclear | 20.2437 | 19.6109 |  |
| Smoking (%) |  |  | <0.001 |
| Now | 13.7441 | 15.8254 |  |
| Ever | 15.9783 | 14.3007 |  |
| Never | 35.8835 | 29.0221 |  |
| Unclear | 34.3940 | 40.8517 |  |
| Activity intensity (%) |  |  | <0.001 |
| Vigorous | 30.9411 | 26.7613 |  |
| Moderate | 21.7332 | 20.0841 |  |
| Minor | 26.2018 | 22.9758 |  |
| Unclear | 21.1239 | 30.1788 |  |
| Prescribed medications (%) |  |  | <0.001 |
| Yes | 62.3561 | 77.3396 |  |
| No | 37.6439 | 22.6078 |  |
| Unclear | 0.0000 | 0.0526 |  |
| Hypertension, n (%) |  |  | <0.001 |
| Yes | 29.4516 | 27.1819 |  |
| No | 42.7894 | 35.6467 |  |
| Unclear | 27.7590 | 37.1714 |  |
| Diabetes (%) |  |  | 0.278 |
| Yes | 11.9838 | 12.7234 |  |
| No | 84.9695 | 85.0683 |  |
| Borderline | 2.9790 | 2.0505 |  |
| Unclear | 0.0677 | 0.1577 |  |
| Close relative with asthma (%) |  |  | <0.001 |
| Yes | 48.0704 | 50.0000 |  |
| No | 44.5498 | 36.4879 |  |
| Unclear | 7.3798 | 13.5121 |  |
| WBC (1000 cells/Ul) | 7.4437 ± 2.6006 | 7.6422 ± 2.3006 | 0.002 |
| LY (1000 cells/Ul) | 2.3713 ± 1.5729 | 2.5286 ± 1.0035 | <0.001 |
| NE (1000 cells/Ul) | 4.1782 ± 1.7481 | 4.1770 ± 1.8457 | 0.535 |
| PLT (1000 cells/Ul) | 254.9391 ± 66.6742 | 270.1519 ± 74.7882 | <0.001 |
| SII | 500.9029 ± 289.5152 | 492.5217 ± 295.3296 | 0.200 |

Mean ± SD for continuous variables: P value was calculated by weighted linear regression model. % For Categorical variables: P value as calculated by weighted chi-square test.

Supplement **Table 4** Characteristics of participants in **DURAIION**

| Characteristics | < 11 years(n=1814) | ≥ 11 years(n=1900) | *P-value* |
| --- | --- | --- | --- |
| Age (years) | 22.9019 ± 22.5110 | 43.9747 ± 20.0804 | <0.001 |
| Gender (%) |  |  | <0.001 |
| Male | 47.6847 | 39.0000 |  |
| Female | 52.3153 | 61.0000) |  |
| Race (%) |  |  | <0.001 |
| White | 30.0441 | 41.7368 |  |
| Black | 31.8633 | 27.6842 |  |
| Other race | 38.0926 | 30.5789 |  |
| Education level (%) |  |  | <0.001 |
| Less than high school | 57.6626 | 30.6316 |  |
| High school | 9.3164 | 20.8421 |  |
| More than high school | 16.6483 | 48.3684 |  |
| Unclear | 16.3727 | 0.1579 |  |
| PIR | 1.8657 ± 1.4147 | 2.1347 ± 1.5284 | <0.001 |
| BMI (kg/m^2^) | 24.6727 ± 8.9201 | 31.0240 ± 8.6447 | <0.001 |
| Alcohol (g), (%) |  |  | <0.001 |
| < 0 | 70.1764 | 63.1579 |  |
| ≥ 0 | 7.2216 | 20.0526 |  |
| Unclear | 22.6020 | 16.7895 |  |
| Smoking (%) |  |  | <0.001 |
| Now | 8.7100 | 21.8421 |  |
| Ever | 8.1036 | 21.7895 |  |
| Never | 18.1367 | 43.1053 |  |
| Unclear | 65.0496 | 13.2632 |  |
| Activity intensity (%) |  |  | <0.001 |
| Vigorous | 19.8456 | 37.0526 |  |
| Moderate | 12.4035 | 29.3684 |  |
| Minor | 16.2624 | 31.4211 |  |
| Unclear | 51.4884 | 2.1579 |  |
| Prescribed medications (%) |  |  | <0.001 |
| Yes | 66.5932 | 74.3684 |  |
| No | 33.3517 | 25.5263 |  |
| Unclear | 0.0551 | 0.1053 |  |
| Hypertension, n (%) |  |  | <0.001 |
| Yes | 17.0893 | 38.8421 |  |
| No | 22.4917 | 54.0000 |  |
| Unclear | 60.4190 | 7.1579 |  |
| Diabetes (%) |  |  | <0.001 |
| Yes | 7.3870 | 17.0000 |  |
| No | 91.0695 | 79.5789 |  |
| Borderline | 1.4884 | 3.2632 |  |
| Unclear | 0.0551 | 0.1579 |  |
| Close relative with asthma (%) |  |  | <0.001 |
| Yes | 45.0386 | 51.2632 |  |
| No | 36.1080 | 45.4211 |  |
| Unclear | 18.8534 | 3.3158 |  |
| WBC (1000 cells/Ul) | 7.6078 ± 2.5713 | 7.5082 ± 2.3599 | 0.326 |
| LY (1000 cells/Ul) | 2.6967 ± 1.4380 | 2.2371 ± 1.0638 | <0.001 |
| NE (1000 cells/Ul) | 3.9599 ± 1.8357 | 4.3915 ± 1.7653 | 0.535 |
| PLT (1000 cells/Ul) | 280.1080 ± 73.6709 | 247.9695 ± 65.5024 | <0.001 |
| SII | 460.9701 ± 286.4726 | 529.7505 ± 291.9195 | <0.001 |

Mean± SD for continuous variables: P value was calculated by weighted linear regression model. % For Categorical variables: P value as calculated by weighted chi-square test.
